# In-hospital Enrollment Into an Electronic Patient Portal Results in Improved Follow-up After Orthopedic Surgery: Cluster Randomized Controlled Trial

**DOI:** 10.2196/37148

**Published:** 2022-08-11

**Authors:** Abhiram R Bhashyam, Mira Bansal, Madeline M McGovern, Quirine M J van der Vliet, Marilyn Heng

**Affiliations:** 1 Massachusetts General Hospital Boston, MA United States; 2 Western University of Health Sciences Pomona, CA United States; 3 Harvard Combined Orthopedic Residency Program Massachusetts General Hospital Boston, MA United States; 4 University Medical Center Utrecht Utrecht Netherlands

**Keywords:** outcomes, orthopedic, electronic health records, surgery, eHealth, patient portals

## Abstract

**Background:**

Electronic patient portal (EPP) use is associated with lower no-show rates and increased patient satisfaction. However, there are disparities in enrollment into these communication platforms.

**Objective:**

We hypothesized that guided inpatient enrollment into an EPP would improve clinical follow-up and EPP use rates for patients who underwent orthopedic surgery compared to the usual practice of providing information in the discharge summary.

**Methods:**

We performed a randomized controlled trial of 229 adult patients who were admitted to the hospital for an orthopedic condition that required a 3-month follow-up visit. Patients were cluster-randomized by week to either the control or intervention group. The control group received information on how to enroll into and use the EPP in their discharge paperwork, whereas the intervention group was actively enrolled and taught how to use the EPP. At 3 months postdischarge, the patients were followed to see if they attended their follow-up appointment or used the EPP.

**Results:**

Of the 229 patients, 83% (n=190) presented for follow-up at 3 months (control: 93/116, 80.2%; intervention: 97/113, 85.8%; *P*=.25). The likelihood of EPP use was significantly higher in the intervention group (control: 19/116, 16.4%; intervention: 70/113, 62%; odds ratio [OR] 8.3, 95% CI 4.5-15.5; *P*<.001). Patients in the intervention group who used the EPP were more likely to present for postsurgical follow-up (OR 3.59, 95% CI 1.28-10.06; *P*=.02).

**Conclusions:**

The inpatient enrollment of patients who underwent orthopedic surgery into an EPP increased EPP use but did not independently result in enhanced follow-up. Patients who were enrolled as inpatients and subsequently used the portal had the highest likelihood of 3-month follow-up.

**Trial Registration:**

ClinicalTrials.gov NCT03431259; https://clinicaltrials.gov/ct2/show/NCT03431259

## Introduction

The proper follow-up and collection of patient-reported outcomes is critical to ensuring successful patient care [1-3]. Traditional clinical outcomes and patient-reported outcomes provide clinicians, institutions, and insurers with valuable, reliable measures of the quality of patient outcomes after surgical intervention and can help improve patients’ overall satisfaction and progress [4-6]. Despite increased policy-driven and financial incentives, orthopedic surgeons struggle to gather this information, because historically, follow-up with patients with orthopedic trauma has been poor [7,8]. Finding new ways to engage patients, ensuring that they follow the schedule, and providing outcome data are important goals for all surgeons [1,9-12].

Previous studies have demonstrated that electronic tools, such as electronic patient portals (EPPs), can be valuable methods of achieving these goals [13-15]. These apps give patients the opportunity to manage their own health, with options to view appointments, renew prescriptions, request authorizations for specialist appointments, and access quality health and wellness information. More recently, patients also have the option to use apps to complete web-based questionnaires [12,16,17].

EPP use is associated with lower no-show rates and increased patient satisfaction. However, it is known that there are disparities in patient enrollment into these communication platforms [18,19]. The decreased enrollment and use of EPPs have been previously associated with demographic (age, language, and race) and treatment factors, but strategies to mitigate these disparities have not yet been assessed. Therefore, in this study, we hypothesized that guided inpatient enrollment into an EPP would improve clinical follow-up and EPP use rates for patients who underwent orthopedic surgery compared to the usual practice of providing information on how to enroll in the discharge summary.

## Methods

### Study Design and Setting

In total, 240 patients presenting to the Massachusetts General Hospital for inpatient orthopedic surgery were prospectively enrolled in this randomized controlled study. The trial used a cluster randomization method. The patients were recruited between February 2018 and February 2019 and followed for 3 months.

### Participants

Members of the research team screened and approached all eligible patients to ask for consent. All patients aged ≥18 years admitted to the hospital for an orthopedic condition with the need for outpatient follow-up were eligible for the study. Patients were excluded if they were unable to consent for themselves, could not communicate in English, or did not possess a smartphone.

### Ethics Approval

Institutional review board approval (IRB 2017P001594) was obtained prior to the initiation of the study, and all patients were given a fact sheet if they consented.

### Description of Experiment, Treatment, or Surgery

Eligible patients were cluster-randomized by week into 2 groups. The control group received information on how to enroll into and use the EPP in their discharge paperwork, whereas the intervention group was actively enrolled and taught how to use the EPP.

### Description of Follow-up Routine

In the period between hospital discharge and follow-up, patients from both groups who were registered in the EPP were requested to fill out a survey on their personal device and received a notification of their upcoming clinic appointment.

### Variables, Outcome Measures, Data Sources, and Bias

For all enrolled patients, their age, gender, race (coded as White vs non-White), zip code, and admission diagnosis or service were recorded. Division of race into White and non-White was done to improve the robustness of the statistical analysis. The median income for each patient was abstracted using the zip code of the patient’s residence based on US census data, and the percentage of patients with an income less than the median state income was calculated [20].

Patients were followed for 3 months to ascertain if they completed their follow-up orthopedic clinic appointment and if they used the EPP to read or send a message with their providers, view a result, or answer a survey during the time period from their discharge to their follow-up.

### Demographics and Description of the Study Population

A total of 229 patients were included (116 patients randomized to the control group and 113 patients randomized to the intervention group). The average patient age was 53.5 (SD 16.4) years. Of the 229 patients, 49.8% (n=114) were male and 16.2% (n=37) were non-White. In total, 31% (n=71) of the patients were admitted for the management of an acute traumatic injury, whereas 9.6% (n=22) were admitted for the treatment of an acute musculoskeletal infection. Demographic characteristics were balanced between the intervention and control groups, suggesting successful randomization (Table 1).

**Table 1:** Descriptive statistics of patient demographics and the test of balance.

Variable			All patients (N=229)		Control (n=116)		Intervention (n=113)		*P* value	
Age (years), mean (SD)			53.5 (16.4)		54.5 (16.7)		52.4 (16.0)		.34^a^	
Gender, male, n (%)			114 (49.8)		61 (52.6)		53 (46.9)		.39^b^	
Median income by zip code (2018; US $), mean (SD)			82,039 (27,091)		80,888 (27,853)		83,221 (26,358)		.52^a^	
Less than the median Massachusetts income, n (%)			120 (52.4)		67 (57.8)		53 (46.9)		.10^b^	
Race, non-White, n (%)			37 (16.2)		17 (14.7)		20 (17.7)		.58^b^	
Injury, n (%)			71 (31)		34 (29.3)		37 (32.7)		.57^b^	
Injury or acute infection, n (%)			93 (40.6)		45 (38.8)		48 (42.5)		.57^b^	
**Subspecialty, n (%)**										.57^b^
	Joints	75 (32.8)		38 (32.8)		37 (32.7)		.93^b^		
	Oncology	9 (3.9)		5 (4.3)		4 (3.5)		N/A^c^		
	Sports or shoulder	10 (4.4)		5 (4.3)		5 (4.4)		N/A		
	Spine	41 (17.9)		23 (19.8)		18 (15.9)		N/A		
	Trauma	94 (41)		45 (38.8)		49 (43.4)		N/A		
**Outcome variable, n (%)**										
	Follow-up at 3 months	190 (83)		93 (80.2)		97 (85.8)		.25^b^		
	Any use of the electronic patient portal	89 (38.9)		19 (16.4)		70 (62)		<.001^b^		

^a^*P* value was obtained from a 2-tailed *t* test with unequal variance.

^b^*P* value was obtained from a chi-squared test or Fisher exact test.

^c^N/A: not applicable.

### Accounting for All Patients

**Figure 1:**
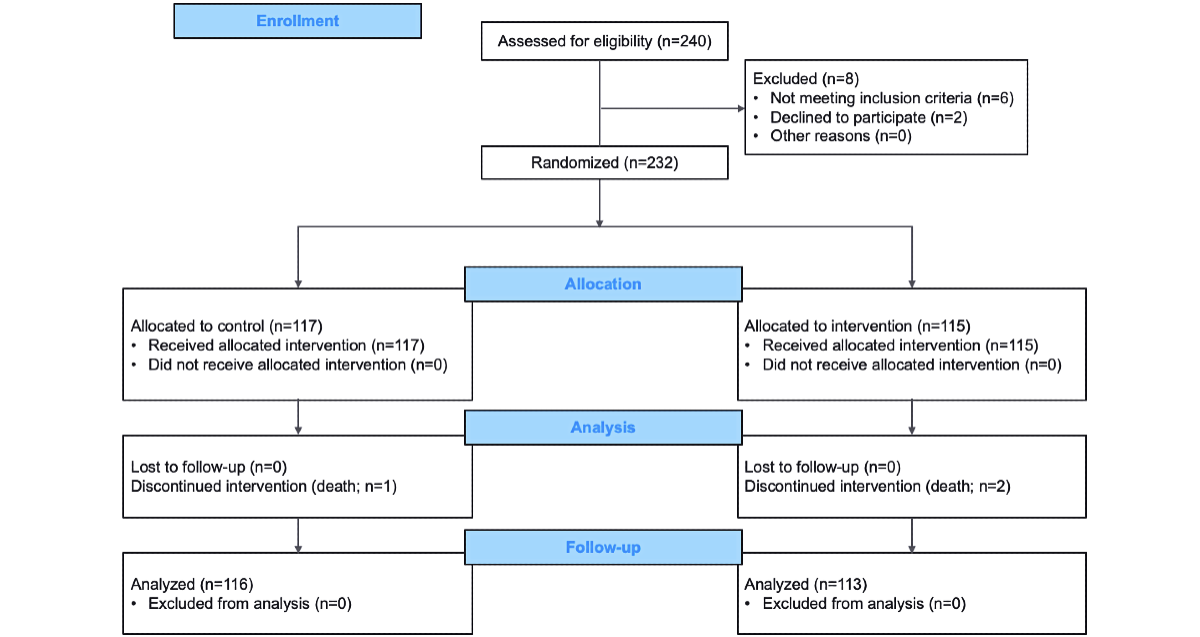
Patient enrollment based on CONSORT (Consolidated Standards of Reporting Trials) flow template.

Patient enrollment is displayed with a flow diagram (Figure 1).

### Statistical Analysis and Study Size

Descriptive statistics were used for the demographic data. Differences between groups were assessed using the chi-square or Fisher exact test for categorical variables and the 2-tailed *t* test or ANOVA for continuous variables. Demographic or treatment factors associated with improved follow-up or EPP use were assessed using forward stepwise logistic regression modeling to avoid overfitting. We also performed a subgroup analysis assessing the effects of the patient’s race and average median income. A robustness analysis exploring the likelihood of enrolling in an EPP or completing follow-up in all patients was also performed. Significance was set at *P*<.05. Stata statistical software (version 14; StataCorp) was used for all analyses.

An a priori power analysis was completed to determine the sample size. We assumed an existing follow-up rate of 70%, and to detect an approximate 10% difference in follow-up with an α of .05, we calculated an approximate sample size of 200 patients distributed equally between both groups.

## Results

Of the 229 patients, 83% (n=190) presented for follow-up at 3 months (control: 93/116, 80.2%; intervention: 97/113, 85.8%; *P*=.25 by chi-square analysis not accounting for interaction effects). In total, 38.9% (89/229) of all patients used the EPP, but use was significantly different between the control and intervention group (control: 19/116, 16.4%; intervention: 70/113, 62%; odds ratio [OR] 8.3, 95% CI 4.5-15.5; *P*<.001; Table 1). Inpatient enrollment into the EPP did not independently result in an increase in the 3-month follow-up rates (OR 1.50, 95% CI 0.75-3.02; *P*=.26; see model 1 in Table 2). Patients who used the EPP were significantly more likely to complete a follow-up visit (OR 3.47, 95% CI 1.46-8.26; *P*=.005; see model 2 in Table 2). In addition, patients in the intervention group who used the EPP were more likely to present for postsurgical follow-up (OR 3.59, 95% CI 1.28-10.06; *P*=.02; see model 3 in Table 2).

**Table 2:** The likelihood of 3-month clinic follow-up based on inpatient enrollment into the electronic patient portal (EPP) with and without interaction effects to account for use of the EPP.

Variable		Model 1, logistic regression without interaction effects, RR^a^ (95% CI)	Model 2, logistic regression without interaction effects, OR^b^ (95% CI)	Model 3, logistic regression with interaction effects, OR (95% CI)	*P* value
Treatment (inpatient enrollment)		1.50 (0.75-3.02)	N/A^c^	N/A	.26
Any use of the EPP		N/A	3.47 (1.46-8.26)	N/A	.005
Interaction effect (inpatient enrollment*any use of the EPP)					
	Control*no use	N/A	N/A	Reference	N/A
	Control*use	N/A	N/A	2.35 (0.50-10.99)	.28
	Intervention*no use	N/A	N/A	0.80 (0.35-1.86)	.61
	Intervention*use	N/A	N/A	3.59 (1.28-10.06)	.02

^a^RR: relative risk.

^b^OR: odds ratio.

^c^N/A: not applicable.

### Subgroup Analysis by Race and Median Income

Among the 229 patients, 28.8% (n=66) of White patients enrolled in the EPP, whereas only 6.1% (n=14) of non-White patients enrolled (*P*=.07 by Fisher exact test). This difference was driven by enrollment disparity in the control group. For non-White patients, only 1 out of 17 in the control group enrolled in the EPP (but did not use it). In contrast, of the 20 non-White patients in the intervention group, 13 (65%) registered and used the EPP (*P*<.001 by Fisher exact test comparing intervention vs control for both groups). Once enrolled, use of the EPP was not statistically different between White and non-White patients (*P*=.81 by Fisher exact test). No statistical differences in EPP registration (*P*>.05), use (*P*>.05), or clinical follow-up (*P*>.05) were observed for median income.

### Robustness Analysis

To compare our results to prior studies on the likelihood of enrolling in an EPP, we performed a backward stepwise logistic regression using measured demographic factors for all patients. We found that older age (OR 0.97, 95% CI 0.95-0.99; *P*=.03) and non-White race (OR 0.13, 95% CI 0.02-1.09; *P*=.06) were associated with decreased odds of EPP enrollment.

## Discussion

### Principal Findings

Tracking patient outcomes following orthopedic surgery is often difficult due to variable and poor follow-up. Electronic apps such as EPPs may be able to bridge this gap by engaging patients following hospital discharge [1,9-12]. In this randomized controlled study, we found that guided inpatient enrollment of patients who underwent orthopedic surgery into an EPP increased EPP use, but this did not independently result in enhanced follow-up. Patients who were enrolled as inpatients and subsequently used the portal had the highest likelihood of 3-month follow-up. In addition, we found that guided inpatient enrollment was associated with increased registration and use of the EPP in non-White patients.

In 2 recent studies of patients who underwent orthopedic surgery, EPP use was associated with lower no-show rates and increased patient satisfaction [18,19]. Both studies also found significant disparities in EPP enrollment based on demographic and treatment factors, but neither assessed strategies to mitigate these disparities [18,19]. Our results suggest that a method to improve the registration and use of EPPs, especially by disadvantaged groups, is to enroll patients while they are still inpatients following surgery. Although this may not independently result in improved follow-up rates, it is a standardized method to improve EPP registration and use for all patients, especially since EPP use is known to improve patient care. For example, a recent systematic review by Schwebel and Larimer [21] demonstrated that SMS text messaging improved patient compliance to appointments, whereas Bigby et al [22] had comparable results through phone calls or manual letters in an outpatient primary care setting.

Multiple retrospective studies have demonstrated that EPP use improves the likelihood of attending follow-up visits [18,19,23]. Using a prospective framework, we also found that EPP enrollment and use was associated with improved follow-up, but simple enrollment in an EPP was not independently associated with improved follow-up. This result suggests that a possible explanation for results in prior retrospective studies between EPP registration and use and enhanced follow-up may be due to patient confounding. Patients who are motivated to register and enroll in an EPP are also more likely to present for clinical follow-up. As in other social interactions frameworks, our findings suggest that patient portal apps may improve follow-up rates and survey completion if some preconditions are met: (1) patients need to be widely exposed and aware of the patient portal and (2) patients need to incorporate the use of the app into their daily routines with relevant content and context (ie, “stickiness” and appropriate context). To reinforce the importance of using these portals to patients, clinicians may need to implement a few changes in their practice. First, someone from the clinical team should enroll patients in the app either while they are still an inpatient or in the outpatient clinics to ensure successful enrollment and an understanding of the app. Next, to make the notifications from the app more readily accessible to patients, there needs to be an update to the app that includes notifications in forms more immediate than email reminders such as SMS text messaging or app push notifications. Finally, surgeons should also encourage communication through the patient portal, so patients feel more motivated to check and use the app.

Finally, in our supplemental analysis, we observed that non-White race was associated with decreased odds of EPP enrollment. For non-White patients, only 1 out of 17 patients in the control group signed up for the EPP, and that patient never used it. In contrast, of the 20 non-White patients in the intervention group, 65% used the EPP. This analysis suggests that standardized enrollment only partially alleviates the barriers to benefits from EPP use. Future studies should further assess the effects of guided enrollment in disadvantaged groups [18].

There were several important limitations to this study that may have impacted the results. First, we specifically approached English-speaking patients with active email addresses and smartphones. If we learned that they did not have either upon interview, we would exclude them from the study. This exclusion criteria may have decreased enrollment from the older patient population as well as patients from lower socioeconomic backgrounds who were less likely to be technologically active, although we attempted to mitigate this in our analysis by including median income by zip code. Future studies are needed to assess the effects of guided inpatient enrollment specifically in disadvantaged groups based on existing literature and our study [18,19]. Based on the post hoc power analysis, our results lacked the statistical power (power=25.6%) to detect no differences in clinical follow-up rates. We may have similarly been limited by the sample size for our subgroup analysis of non-White race, although our sample estimates are proportional to state population statistics [20]. We also referred to the non-White subgroup as disadvantaged not due to race alone but other socioeconomic features measured in our data set. Therefore, although this can be generalized in aggregate, it may not be true for any single patient. With a larger sample size, it may be that guided enrollment, especially for some patient populations, would have statistically and clinically relevant differences in follow-up rates.

### Conclusions

The inpatient enrollment of patients who underwent orthopedic surgery into an EPP increased EPP use, but this did not independently result in enhanced follow-up. Patients who were enrolled as inpatients and subsequently used the portal had the highest likelihood of 3-month follow-up. Future studies targeted toward disadvantaged groups are critically needed.

## Appendix

Multimedia Appendix 1 CONSORT-eHEALTH checklist (V 1.6.2).

## Abbreviations

EPP: electronic patient portal
OR: odds ratio
