# Supplementary material for: In-hospital Enrollment Into an Electronic Patient Portal Results in Improved Follow-up After Orthopedic Surgery: Cluster Randomized Controlled Trial
Source: JMIR Perioper Med. 2022 Aug 11;5(1):e37148. doi: 10.2196/37148 (PMC9412718; doi:10.2196/37148)
Supplement: Multimedia Appendix 1 [file periop_v5i1e37148_app1.pdf]

CONSORT-EHEALTH Checklist V1.6.2 Report	Manuscript Number	37148
**(based on CONSORT-EHEALTH V1.6), available at [http://tinyurl.com/consort-ehealth-v1-6].**		
**Date completed**		
3/9/2022 0:49:44		
**by**		
Mira Bansal		
In-hospital enrollment into an electronic patient portal results in improved follow-up after orthopedic surgery: Cluster Randomized Trial		
TITLE		
**1a-i) Identify the mode of delivery in the title**		
"Electronic patient portal (EPP) use is associated with lower no-show rates and increased patient satisfaction." "Inpatient enrollment of orthopaedic surgery patients into an EPP increased EPP use, but this did not independently result in enhanced follow-up."		
**1a-ii) Non-web-based components or important co-interventions in title**		
**1a-iii) Primary condition or target group in the title**		
"We performed a randomized controlled trial of 229 adult patients who were admitted to the hospital for an orthopedic condition that required a three-month follow-up visit."		
ABSTRACT		
**1b-i) Key features/functionalities/components of the intervention and comparator in the METHODS section of the ABSTRACT**		
"We performed a randomized controlled trial of 229 adult patients who were admitted to the hospital for an orthopedic condition that required a three-month follow-up visit. Patients were cluster randomized by week to either the control or intervention group. The control received information on how to enroll into and use the EPP in their discharge paperwork, while the intervention was actively enrolled and taught. At 3 months post-discharge, patients were followed to see if they attended their follow-up appointment or used the EPP. "		
**1b-ii) Level of human involvement in the METHODS section of the ABSTRACT**		
**1b-iii) Open vs. closed, web-based (self-assessment) vs. face-to-face assessments in the METHODS section of the ABSTRACT**		
**1b-iv) RESULTS section in abstract must contain use data**		
**1b-v) CONCLUSIONS/DISCUSSION in abstract for negative trials**		
INTRODUCTION		
**2a-i) Problem and the type of system/solution**		
"EPP use is associated with lower no-show rates and increased patient satisfaction. However, it is known that there are disparities in patient enrollment into these communication platforms.[18, 19] Decreased enrollment and use of EPPs have been previously associated with demographic factors (age, language, race) and treatment factors, but strategies to mitigate these disparities have not yet been assessed."		
**2a-ii) Scientific background, rationale: What is known about the (type of) system**		
"Previous studies have demonstrated that electronic tools, like electronic patient portals (EPP), can be a valuable method of achieving this goal.[13-15] These applications give patients the opportunity to manage their own health with options to view appointments, renew prescriptions, request authorizations for specialists' appointments, and access quality health and wellness information. More recently, patients also have the option to use electronic applications to complete online questionnaires.[12, 16, 17]"		
**Does your paper address CONSORT subitem 2b?**		

"Therefore, in this study, we hypothesized that guided inpatient enrollment into an EPP would improve clinical follow-up and EPP use rates for orthopaedic surgery patients compared to the usual practice of providing information on how to enroll in the discharge summary."		
METHODS		
**3a) CONSORT: Description of trial design (such as parallel, factorial) including allocation ratio**		
"240 patients presenting to Massachusetts General Hospital for inpatient orthopedic surgery were prospectively enrolled in this randomized controlled study." "Eligible patients were cluster randomized by week into two groups. The control group received information on how to enroll into and use the EPP in their discharge paperwork, while the intervention group was actively enrolled and taught how to use the EPP."		
**3b) CONSORT: Important changes to methods after trial commencement (such as eligibility criteria), with reasons**		
We did not have to change the methods after the trial began.		
**3b-i) Bug fixes, Downtimes, Content Changes**		
**4a) CONSORT: Eligibility criteria for participants**		
"Members of the research team screened and approached all eligible patients to ask for consent. All patients age 18 years or older admitted to the hospital for an orthopedic condition with the need for outpatient follow-up were eligible for the study. Patients were excluded if they were unable to consent for themselves, could not communicate in English, and did not possess a smartphone. Institutional review board approval was obtained prior to the initiation of the study, and all patients were given a Fact Sheet if they consented."		
**4a-i) Computer / Internet literacy**		
**4a-ii) Open vs. closed, web-based vs. face-to-face assessments:**		
"Members of the research team screened and approached all eligible patients to ask for consent. All patients age 18 years or older admitted to the hospital for an orthopedic condition with the need for outpatient follow-up were eligible for the study. Patients were excluded if they were unable to consent for themselves, could not communicate in English, and did not possess a smartphone."		
**4a-iii) Information giving during recruitment**		
**4b) CONSORT: Settings and locations where the data were collected**		
"240 patients presenting to Massachusetts General Hospital for inpatient orthopedic surgery were prospectively enrolled in this randomized controlled study. The trial utilized a cluster randomization method. Patients were recruited between February 2018 and February 2019 and followed for three months." "In the period between hospital discharge and follow-up, patients from both groups that were registered in the EPP were requested to fill out a survey on their personal device and also received a notification of their upcoming clinic appointment."		
**4b-i) Report if outcomes were (self-)assessed through online questionnaires**		
"In the period between hospital discharge and follow-up, patients from both groups that were registered in the EPP were requested to fill out a survey on their personal device and also received a notification of their upcoming clinic appointment." "Patients were followed for three months to ascertain if they completed their follow-up orthopedic clinic appointment and if they used the EPP to read or send a message with their providers, view a result, or answer a survey during the time period from their discharge to their follow-up."		
**4b-ii) Report how institutional affiliations are displayed**		
**5) CONSORT: Describe the interventions for each group with sufficient details to allow replication, including how and when they were actually administered**		
**5-i) Mention names, credential, affiliations of the developers, sponsors, and owners**		
**5-ii) Describe the history/development process**		
**5-iii) Revisions and updating**		

**5-iv) Quality assurance methods**		
**5-v) Ensure replicability by publishing the source code, and/or providing screenshots/screen-capture video, and/or providing flowcharts of the algorithms used**		
**5-vi) Digital preservation**		
**5-vii) Access**		
Participants were required to have a smart phone on which they could access the internet.		
**5-viii) Mode of delivery, features/functionalities/components of the intervention and comparator, and the theoretical framework**		
"Previous studies have demonstrated that electronic tools, like electronic patient portals (EPP), can be a valuable method of achieving this goal.[13-15] These applications give patients the opportunity to manage their own health with options to view appointments, renew prescriptions, request authorizations for specialists' appointments, and access quality health and wellness information. More recently, patients also have the option to use electronic applications to complete online questionnaires.[12, 16, 17]" "Patients were followed for three months to ascertain if they completed their follow-up orthopedic clinic appointment and if they used the EPP to read or send a message with their providers, view a result, or answer a survey during the time period from their discharge to their follow-up."		
**5-ix) Describe use parameters**		
**5-x) Clarify the level of human involvement**		
**5-xi) Report any prompts/reminders used**		
"In the period between hospital discharge and follow-up, patients from both groups that were registered in the EPP were requested to fill out a survey on their personal device and also received a notification of their upcoming clinic appointment."		
**5-xii) Describe any co-interventions (incl. training/support)**		
"Eligible patients were cluster randomized by week into two groups. The control group received information on how to enroll into and use the EPP in their discharge paperwork, while the intervention group was actively enrolled and taught how to use the EPP."		
**6a) CONSORT: Completely defined pre-specified primary and secondary outcome measures, including how and when they were assessed**		
"For all enrolled patients, their age, gender, race (coded as White versus non-White), zip-code and admission diagnosis/service were recorded. The median income for each patient was abstracted using the zip code of the patient's residence based on U.S. Census data, and the percentage of patients with income less than the median state income was calculated.[20]<br>Patients were followed for three months to ascertain if they completed their follow-up orthopedic clinic appointment and if they used the EPP to read or send a message with their providers, view a result, or answer a survey during the time period from their discharge to their follow-up."		
**6a-i) Online questionnaires: describe if they were validated for online use and apply CHERRIES items to describe how the questionnaires were designed/deployed**		
**6a-ii) Describe whether and how "use" (including intensity of use/dosage) was defined/measured/monitored**		
**6a-iii) Describe whether, how, and when qualitative feedback from participants was obtained**		
**6b) CONSORT: Any changes to trial outcomes after the trial commenced, with reasons**		

"240 patients presenting to Massachusetts General Hospital for inpatient orthopedic surgery were prospectively enrolled in this randomized controlled study. The trial utilized a cluster randomization method. Patients were recruited between February 2018 and February 2019 and followed for three months." "In the period between hospital discharge and follow-up, patients from both groups that were registered in the EPP were requested to fill out a survey on their personal device and also received a notification of their upcoming clinic appointment."		
**7a) CONSORT: How sample size was determined**		
**7a-i) Describe whether and how expected attrition was taken into account when calculating the sample size**		
**7b) CONSORT: When applicable, explanation of any interim analyses and stopping guidelines**		
"For all enrolled patients, their age, gender, race (coded as White versus non-White), zip-code and admission diagnosis/service were recorded. The median income for each patient was abstracted using the zip code of the patient's residence based on U.S. Census data, and the percentage of patients with income less than the median state income was calculated.[20]<br>Patients were followed for three months to ascertain if they completed their follow-up orthopedic clinic appointment and if they used the EPP to read or send a message with their providers, view a result, or answer a survey during the time period from their discharge to their follow-up."		
**8a) CONSORT: Method used to generate the random allocation sequence**		
"Eligible patients were cluster randomized by week into two groups.		
**8b) CONSORT: Type of randomisation; details of any restriction (such as blocking and block size)**		
"Eligible patients were cluster randomized by week into two groups. The control group received information on how to enroll into and use the EPP in their discharge paperwork, while the intervention group was actively enrolled and taught how to use the EPP."		
**9) CONSORT: Mechanism used to implement the random allocation sequence (such as sequentially numbered containers), describing any steps taken to conceal the sequence until interventions were assigned**		
The research assistant presented the study to the patient and only shared patient's randomization after they consented to participate.		
**10) CONSORT: Who generated the random allocation sequence, who enrolled participants, and who assigned participants to interventions**		
Members of the research team		
**11a) CONSORT: Blinding - If done, who was blinded after assignment to interventions (for example, participants, care providers, those assessing outcomes) and how**		
**11a-i) Specify who was blinded, and who wasn't**		
Both research staff and patients were not blinded.		
**11a-ii) Discuss e.g., whether participants knew which intervention was the "intervention of interest" and which one was the "comparator"**		
**11b) CONSORT: If relevant, description of the similarity of interventions**		
"The control group received information on how to enroll into and use the EPP in their discharge paperwork, while the intervention group was actively enrolled and taught how to use the EPP."		
**12a) CONSORT: Statistical methods used to compare groups for primary and secondary outcomes**		
"Descriptive statistics were used for demographic data. Differences between groups were assed using the chi-square or Fisher's exact test for categorical variables and the t-test and/or analysis of variance (ANOVA) for continuous variables. Demographic or treatment factors associated with improved follow-up or EPP use were assessed using forward stepwise logistic regression modeling to avoid overfitting. We also performed a sub-group analysis assessing the the effect of patient race and average median income. A robustness analysis exploring the likelihood of enrolling in an EPP or completing follow-up in all patients was also performed. Significance was set at P < 0.05. Stata software, version 14 (StataCorp), was used for all analyses.<br><br>An a priori power analysis was completed to determine sample size, we assumed an existing follow-up rate of 70%, and to detect an ~10 % difference in follow-up with an alpha of 0.05, we calculated an approximate sample size of 200 patients distributed equally between both groups."		
**12a-i) Imputation techniques to deal with attrition / missing values**		
"A robustness analysis exploring the likelihood of enrolling in an EPP or completing follow-up in all patients was also performed. Significance was set at P < 0.05. Stata software, version 14 (StataCorp), was used for all analyses."		
**12b) CONSORT: Methods for additional analyses, such as subgroup analyses and adjusted analyses**		

"We also performed a sub-group analysis assessing the the effect of patient race and average median income. A robustness analysis exploring the likelihood of enrolling in an EPP or completing follow-up in all patients was also performed. Significance was set at P < 0.05. Stata software, version 14 (StataCorp), was used for all analyses"		
RESULTS		
**13a) CONSORT: For each group, the numbers of participants who were randomly assigned, received intended treatment, and were analysed for the primary outcome**		
"Including all patients, 83% presented for follow-up at 3 months (80.2 vs 85.8, control vs intervention, P=0.254 by chi-square analysis not accounting for interaction effects). In total, 38.9% of all patients used the EPP, but use was significantly different between the control and intervention group (16.4% vs 62.0%; OR 8.3, 95% confidence interval = 4.5, 15.5; P<0.001, Table 1). Inpatient enrollment into the EPP did not independently result in an increase in 3-month follow-up rates (OR 1.50, 95% CI = 0.75, 3.02; p=0.256, Table 2-Model 1). Patients who used the EPP were significantly more likely to complete a follow-up visit (OR 3.47, 95% confidence interval =1.46, 8.26; P=0.005, Table 2-Model 2). In addition, intervention patients who used the EPP were more likely to present for post-surgical follow-up (OR 3.59, 95% confidence interval = 1.28, 10.06; P = 0.015, Table 2-Model 3)."		
**13b) CONSORT: For each group, losses and exclusions after randomisation, together with reasons**		
Included in figure 1. 8 participants were ultimately excluded.		
**13b-i) Attrition diagram**		
**14a) CONSORT: Dates defining the periods of recruitment and follow-up**		
"Patients were recruited between February 2018 and February 2019 and followed for three months."		
**14a-i) Indicate if critical “secular events” fell into the study period**		
**14b) CONSORT: Why the trial ended or was stopped (early)**		
The trial ended after target enrollment was reached.		
**15) CONSORT: A table showing baseline demographic and clinical characteristics for each group**		
Table 1: Descriptive Statistics of Patient Demographics and Test of Balance; *t-test w/ unequal variance, ¥Chi-squared test or Fisher’s exact test		
**15-i) Report demographics associated with digital divide issues**		
"We found that older age (OR 0.97, 95% confidence interval = 0.95, 0.99, P=0.028) and non-White race (OR 0.13, 95% confidence interval 0.02, 1.09, P=0.06) were associated with decreased odds of EPP enrollment."		
**16a) CONSORT: For each group, number of participants (denominator) included in each analysis and whether the analysis was by original assigned groups**		
**16-i) Report multiple “denominators” and provide definitions**		
"Among all patients, 28.7% of White patients enrolled in the EPP while only 6.25% of non-White patients enrolled (P=0.066). This difference was driven by enrollment disparity in the control group. For non-White patients, only 1 out of 16 patients in the control group enrolled in the EPP (but did not use it). In contrast out of the 20 non-White patients in the treatment group, 13 patients (65%) registered and used the EPP (P<0.001, Table 3). Once enrolled, use of the EPP was not statistically different between White and non-White Patients (Table 3). No statistical differences in EPP registration, use or clinical follow-up were observed for median income."		
**16-ii) Primary analysis should be intent-to-treat**		
**17a) CONSORT: For each primary and secondary outcome, results for each group, and the estimated effect size and its precision (such as 95% confidence interval)**		
"Including all patients, 83% presented for follow-up at 3 months (80.2 vs 85.8, control vs intervention, P=0.254 by chi-square analysis not accounting for interaction effects). In total, 38.9% of all patients used the EPP, but use was significantly different between the control and intervention group (16.4% vs 62.0%; OR 8.3, 95% confidence interval = 4.5, 15.5; P<0.001, Table 1). Inpatient enrollment into the EPP did not independently result in an increase in 3-month follow-up rates (OR 1.50, 95% CI = 0.75, 3.02; p=0.256, Table 2-Model 1). Patients who used the EPP were significantly more likely to complete a follow-up visit (OR 3.47, 95% confidence interval =1.46, 8.26; P=0.005, Table 2-Model 2). In addition, intervention patients who used the EPP were more likely to present for post-surgical follow-up (OR 3.59, 95% confidence interval = 1.28, 10.06; P = 0.015, Table 2-Model 3)."		

**17a-i) Presentation of process outcomes such as metrics of use and intensity of use**		
**17b) CONSORT: For binary outcomes, presentation of both absolute and relative effect sizes is recommended**		
"To compare our results to prior studies on the likelihood of enrolling in an EPP, we performed a backward stepwise logit regression using measured demographic factors for all patients. We found that older age (OR 0.97, 95% confidence interval = 0.95, 0.99, P=0.028) and non-White race (OR 0.13, 95% confidence interval 0.02, 1.09, P=0.06) were associated with decreased odds of EPP enrollment."		
**18) CONSORT: Results of any other analyses performed, including subgroup analyses and adjusted analyses, distinguishing pre-specified from exploratory**		
"Among all patients, 28.7% of White patients enrolled in the EPP while only 6.25% of non-White patients enrolled (P=0.066). This difference was driven by enrollment disparity in the control group. For non-White patients, only 1 out of 16 patients in the control group enrolled in the EPP (but did not use it). In contrast out of the 20 non-White patients in the treatment group, 13 patients (65%) registered and used the EPP (P<0.001, Table 3). Once enrolled, use of the EPP was not statistically different between White and non-White Patients (Table 3). No statistical differences in EPP registration, use or clinical follow-up were observed for median income."		
**18-i) Subgroup analysis of comparing only users**		
**19) CONSORT: All important harms or unintended effects in each group**		
No specific harm or unintended effects to disclose		
**19-i) Include privacy breaches, technical problems**		
**19-ii) Include qualitative feedback from participants or observations from staff/researchers**		
DISCUSSION		
**20) CONSORT: Trial limitations, addressing sources of potential bias, imprecision, multiplicity of analyses**		
**20-i) Typical limitations in ehealth trials**		
"There were several important limitations to this study that may have impacted the results. Firstly, we specifically approached English-speaking patients with active email addresses and those who had smart phones. Upon conversation if we learned that they did not have either, we would exclude them from the study. This may have decreased enrollment from older patient population as well as patients from lower socioeconomic backgrounds who were less likely to be technologically active, although we attempted to mitigate this in our analysis by including median income by zip code." "We may have similarly been limited in sample size for our sub-group analysis of non-White race, although our sample estimates are proportional to state population statistics.[20] We also referred to the non-White subgroup as disadvantaged not due to race alone but other socioeconomic features measured in our dataset. Therefore, while this can be generalized in aggregate, it may not be true for any single patient. With a larger sample size, it may be that guided enrollment, especially for some patient populations, would have statistically and clinically relevant differences in follow-up rates."		
**21) CONSORT: Generalisability (external validity, applicability) of the trial findings**		
**21-i) Generalizability to other populations**		
**21-ii) Discuss if there were elements in the RCT that would be different in a routine application setting**		
**22) CONSORT: Interpretation consistent with results, balancing benefits and harms, and considering other relevant evidence**		
**22-i) Restate study questions and summarize the answers suggested by the data, starting with primary outcomes and process outcomes (use)**		
"Tracking patient outcomes following orthopaedic surgery is often difficult due to variable and poor follow-up. Electronic apps like EPPs may be able to bridge this gap by engaging patients following hospital discharge.[1, 9-12] In this randomized controlled study, we found that guided inpatient enrollment of orthopaedic surgery patients into an EPP increased EPP use, but this did not independently result in enhanced follow-up. Patients who were enrolled as inpatients and subsequently used the portal had the highest likelihood of 3-month follow-up. In addition, we found that guided inpatient enrollment was associated with increased registration and use of an EPP in non-White patients."		

**22-ii) Highlight unanswered new questions, suggest future research**		
Other information		
**23) CONSORT: Registration number and name of trial registry**		
ClinicalTrials.gov Identifier: NCT03431259		
**24) CONSORT: Where the full trial protocol can be accessed, if available**		
I do not believe It is available.		
**25) CONSORT: Sources of funding and other support (such as supply of drugs), role of funders**		
No sources of funding to disclose.		
**X26-i) Comment on ethics committee approval**		
**x26-ii) Outline informed consent procedures**		
**X26-iii) Safety and security procedures**		
**X27-i) State the relation of the study team towards the system being evaluated**		
